# Fem and Von Mises Analysis of OSSTEM ^®^ Dental Implant Structural Components: Evaluation of Different Direction Dynamic Loads

**DOI:** 10.2174/1874210601812010219

**Published:** 2018-03-30

**Authors:** Gabriele Cervino, Umberto Romeo, Floriana Lauritano, Ennio Bramanti, Luca Fiorillo, Cesare D’Amico, Dario Milone, Luigi Laino, Francesco Campolongo, Silvia Rapisarda, Marco Cicciù

**Affiliations:** 1 Department of Biomedical and Dental Sciences, Morphological and Functional Images, School of Dentistry, University of Messina, ME, Italy; 2 Department of Oral and Maxillo-facial Sciences, Pediatric Dentistry Unit, “Sapienza” University of Rome, Rome, Italy; 3 Departments of Engineering, University of Messina, Messina, Italy; 4 Multidisciplinary Department of Medical-Surgical and Odontostomatological Specialties, University of Campania “Luigi Vanvitelli”, Naples, NA, Italy; 5 Micerium Implantology, Micerium SPA, Avengno, Liguria, GE, Italy

**Keywords:** Fixture, Finite element analysis, Dental prosthesis, Implant-supported, Abutment-implant screw, Osteointegration

## Abstract

**Purpose:**

The objective of this investigation is to study prosthodontics and internal components resistance to the masticatory stress and considering different force directions by using Finite Element Method analysis (FEM). The structural materials of the components are usually Titanium alloy grade 4 or 5 and thus, guarantee the integration of the fixture in the bone due to the osteointegration phenomena. Even if the long-term dental implant survival rate is easy to be obtained and confirmed by numerous researches, the related clinical success, due to the alteration of the mechanical and prosthodontics components is still controversial.

**Methods:**

By applying engineering systems of investigations like FEM and Von Mises analyses, it has been investigated how dental implant material was held against the masticatory strength during the dynamic masticatory cycles. A three-dimensional system involved fixture, abutment and the connection screws, which were created and analyzed. The elastic features of the materials used in the study were taken from recent literature data.

**Results:**

Data revealed a different response for both types of devices, although implant neck and dental abutment showed better results for all conditions of loading while the abutment screw represented aweak point of the system.

**Conclusion:**

The data of this virtual model showed all the features of different prosthetic retention systems under the masticatory load. Clinicians should find better prosthetic balance in order to better distribute the stress over the component and to guarantee patients’ clinical long-term results.

## 1 INTRODUCTION

Dental implant procedures have become increasingly effective in the field of oral rehabilitation with numerous published clinical studies recording about the 99% of long-term survival rate. However, several trials regarding the prosthetic complications of the dental implants highlighted how abutments screw loosening, abutment screw fracture, and the implant mid-body breaks may occur frequently. This event especially happens in single dental crowns, which do not distribute the bite force to other implants. In the common two-piece implant systems, the abutment is connected to the implant mechanically by using an internal abutment-implant screw [1-4].

This situation creates an interface through which leakage or break may occur during masticatory events. Implant-abutment connections have evolved greatly in an attempt to minimize these complications. There are several dental implant systems, different by shape, connection and geometry and still today, there is no ideal abutment fixture connection. Fracture or break of prosthodontics elements,, however, is not isolated to the prosthetic components and may also occur in internal, platform switching, pure conical or hybrid connection designs as well; however, the quantity of leakage is unknown in both the design types [3-7].

Several published papers recorded the mechanical failures of osteointegrated fixture more due to repeatable stress than fracture strength due to a single static stress. Therefore, static damage of implants components from overload may be influenced by other parameters such as clenching habit, hard food, and premature contact resulting from the failure of occlusal adjustment or planning [8, 9].

Recently manufacturers have developed different types of new surface design and implant-abutment connections shape to have quick osteointegration and long-term prosthodontics device placed over the dental implants. To have long-term clinical success, it is fundamental that the fixture devices have a suitable structure to manage stress while chewing and distribute stress to the peri-implant bone. Other requirements are biocompatibility of components, correct diagnosis, and appropriate surgical and prosthodontics procedures for implant placement. Mandibular movement should be well known by the clinicians and prosthodontics, and the dental implant rehabilitation should guarantee stability and safety under over-load and for a long time [10-12].

The shape and the geometry of the fixture and the abutment system connection with the properties of the material should be considered and well-known by the prosthodontics and clinicians. Numerous computer systems are today able to recreate a perfect simulation of the jaws movement and to have a model fit with the clinician’s daily practice [1, 3, 7, 13, 14].

Finite Element Model (FEM) is a computer method for stress distribution analysis. The effect of loading strengths over dental implant elements and peri-implant bone can be recorded by applying the equivalent stress (von Mises stress), expressed in Megapascals (MPa). The difference of tension distribution is usually presented by different colors and red shows the maximum stress [2, 4, 7].

To the best of our knowledge, the effectiveness of all the dental implant components stressed by dynamic load has been investigated in some previous researches. However, most of them directed the objective of evaluating the static force stressed to dental implant prosthesis and the surrounding bone tissue. Therefore, the purpose of the present investigation was to check the value recorded over a single crown dental implant component subjected to dynamic stress. Since, there are no similar studies evaluating dynamic stress, the purpose of this study was also to consider stress distribution around dental implants, bone and prosthetic component by 3D models.

## 2 MATERIALS AND METHODS

The investigation was performed on single tooth dental implant (Osstem Dental Implant TSIII ^®^) and prosthetic elements of retention in order to point out the possible failure related to the fracture of structural components or to overload on bone tissue. The FEM assists the field dental prosthodontics to better evaluate the mechanical features of each implant-prosthetic component, its physic-chemical and optimal environmental conditions and its close relationship with hard and soft tissues. The homogeneous distribution of the tensional forces developed over dental devices during the masticatory cycles is influenced not only by the number and the position of the dental implants, but also by the structural material, shape, and the diameter of the individual components’ geometry [13-16]. In this study, we evaluated the stress over single crown dental implants.

Key parameters that influence the accuracy of the results of the FEM system should be underlined. Among these are:

- Detailed geometry of the system and the surrounding bone to be modeled.
- Boundary conditions and constraints.
- Material properties.
- Load conditions - Repeated on time-related to masticatory cycle.
- Bone-Implant interface.
- Test of convergence.
- Validation of the model.

The space geometry and shape of the model finite element should reflect the clinical reality as precisely as possible to get biomechanical plausible results. Solid models of the jaw arches, the dental implants and the prosthetic crowns were constructed from CT images and then processed through a CAD (Computer-Aided Design) in 3D FEM. The informatics programs were used in order to recreate the virtual three-dimensional CAD model. Using Corel Draw, the reverse engineering ^®^ was performed. It is a powerful vector graphics software, through which it was possible to rebuild and scale sections of the facility object of study. The reconstruction of the three-dimensional model was performed in ANSYS Workbench, using the vector geometries output from Corel Draw. The analysis process was then divided into two phases: Pre-processing; construction phase of the finite element model and Post-processing; processing and representation of the solutions [1, 5, 13].

### 2.1 Reverse Engineering and CAD Model

**Fig. (1):**
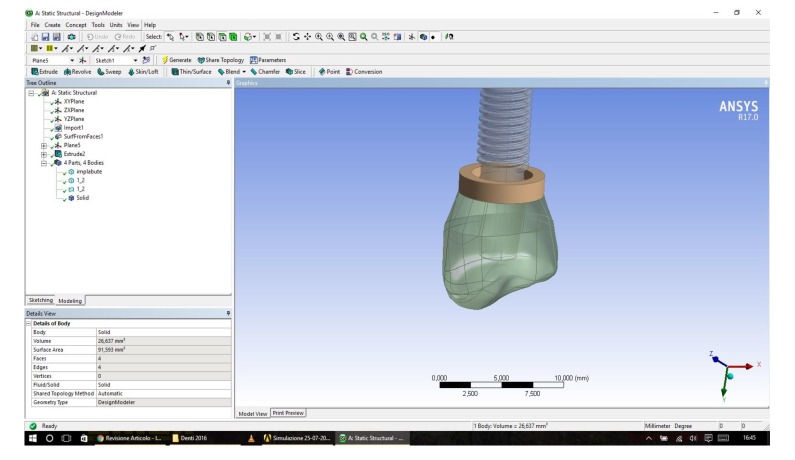
Creating a three-dimensional model of the dental implant with a single crown.

**Fig. (2):**
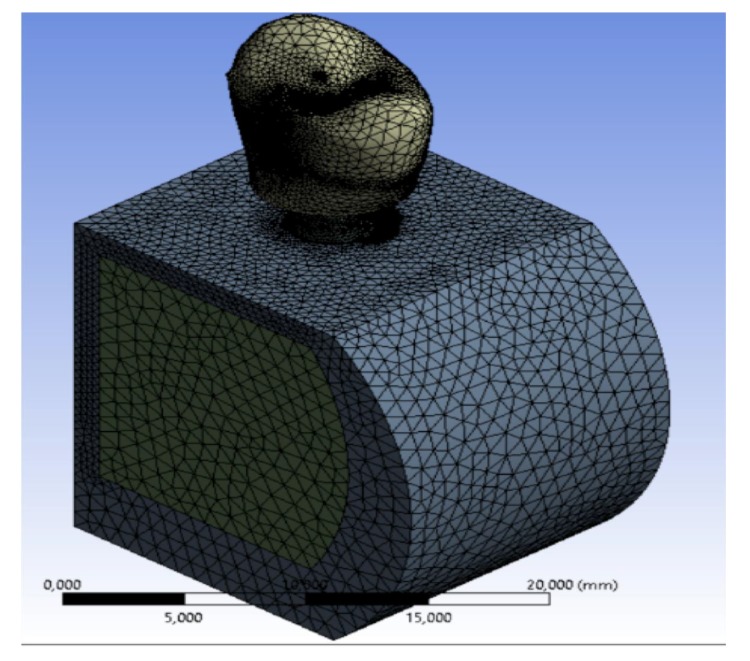
The mesh created for the model with the characteristic of each material added in the model. Different color for cortical and mid collar bone as for the dental implant components and crown.

**Fig. (3):**
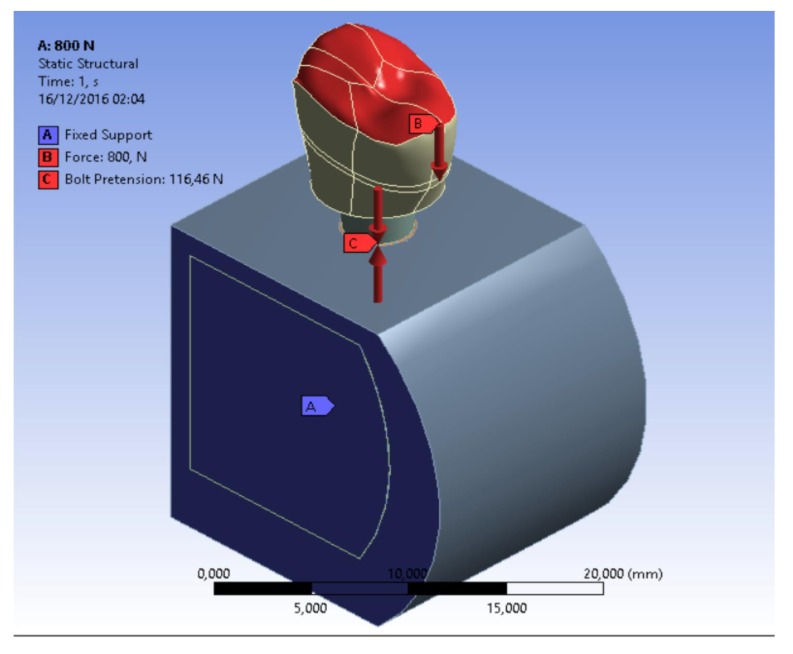
The load involved in the study. A force of 800 Nmm repeated for 2000 times in order to simulate the dynamic stress of the masticatory cycle.

**Fig. (4):**
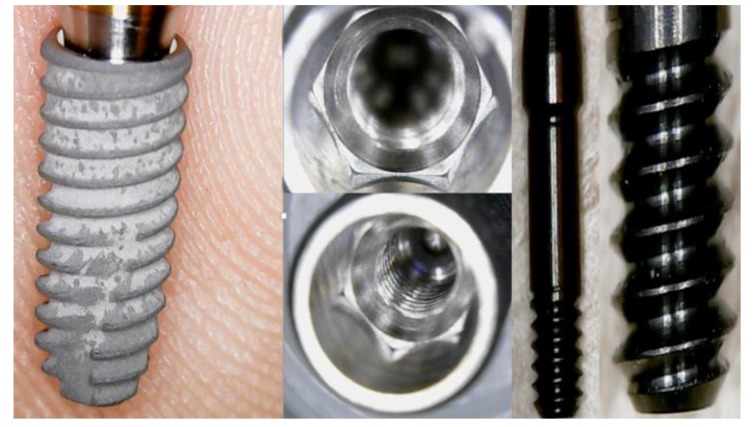
Dental implant components used in the study. Fixture and abutment made on titanium alloy 4 and abutment screw on black gold alloy.

In our created models, both the dental implants with all the components were recreated by using Corel Draw Graphic Suite X7 (Figs. **1**-**4**).

The dimensions were realized from the implant-prosthetic components and made real by the small details of their physic-chemical characteristics provided by the scientific literature and catalogues of the used brand (Osstem Dental Implant TSIII regular ^®^ MICERIUM, ITALY). Through the modeling phase, the information was passed from the physical system to a mathematical model, extrapolating from the same number of variables and ” filtering out ” the remaining ones. It was performed using Ansys Workbench ®. The implant prosthetic threads were properly reshaped in order to recreate the fixture geometry surface.

### 2.2 The Finite Element Analysis

Then, after obtaining these models, three-dimensional CAD, the FEA jaw -implant - prosthesis was performed with ANSYS WORKBENCH 15.0 ^®^, program characterized by a bi-directional connectivity CAD, by high productivity and by an innovative design vision that binds the entire simulation process.

A 3D linear static structural simulation was performed showing the relation (stress and strain) between bone and implant prosthodontics elements; fixture, abutment and the abutment screw. A lower premolar single crown placed over dental implant was simulated in order to replace the mandibular chewing movements in lateral and occlusal directions.

### 2.3 Choice of Materials

In this experimental study, titanium grade 4 is the material used in the construction of the dental fixture, abutment and for all the prosthetics components, while the abutment connection screw was considered in gold alloy according to manufacturing indications.

The properties of materials have been specified in terms of Young's modulus, Poisson's ratio and density. The different physical behavior of the materials stressed by the occlusal loads and lateral forces has been considered. It was hard to reproduce the temporomandibular movement; for this reason, it was chosen to apply a high force to stress and overload the system and the stress was repeated for about 2000 times.

Value of the materials involved in the study:

- The titanium alloys have a limit of resistance for at least 5 times greater than that of the ceramic which can be subjected to a voltage of up to 1000 MPa (equivalent to 1000 kg on each mm2 of the surface) and do not involve rupture of the crash, or fractures per pulse.
- The golden alloy (abutment connection screw) has a a medium value of tension, but lower than titanium alloy [1, 12, 15].
- The bones’ reproduction was considered orthotropic (both for the cortical bone and the cancellous bone); the reference values ​​were taken from the literature and it was divided into cortical and mid collar bone [1-6].
- The dental crown was recreated in Zirconium Ceramic and the fixture was placed into both cortical and mid collar bone. Table **1** shows the characteristics of resistance and elasticity, which are considered for each component of the model.

### 2.4 Creating the Correct MESH

**Fig. (5):**
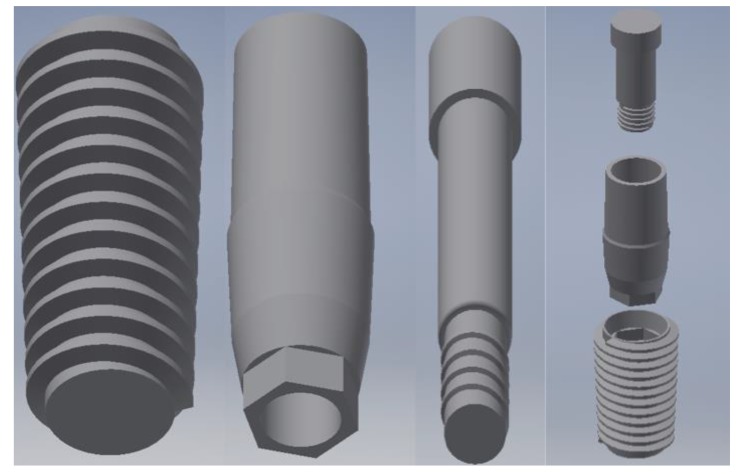
The system reproduced the dental implant components and the stress was directed over each section.

The discretization of the geometry aimed to obtain a discrete model of a continuous object. It is structured in a finite number of freedom degrees (meshing). A polygonal mesh is a collection of vertices, edges and faces that define the shape of a polyhedral object in 3D computer graphics and solid modeling (Figs. **1**-**5**).

Using the programs “SOLID186”, SOLID187 of the ANSYS library, discretization was performed. A maximum gap of about 0.8, with a standard deviation of about 0.16 for all the models showed a high-quality mesh (Figs. **1**, **2**).

### 2.5 Loading Conditions and Dynamic Stress

The components of dental implants were tested with a straight inclination of 30° and with dynamic loads 2000 times each. Different loading conditions were considered. All loads were distributed on the implant surface components in contact with the tooth crown.

### 2.6 Constrain Conditions and Contacts

The bone-implant and the bone-bone contact conditions are considered as bonded. It was simulated as the ideal osseointegration with total contact surface between the implant and the bone (Bone-Implant Contact - BIC), with no possibility scroll between the two areas. Moreover, for all the threaded connections, a bolt pretension was considered in accordance with the installation requirements; in particular implant/bone=46,5N; abutment/implant = 50N; bolt/implant = 62,5N (Table **1**).

## 3 SIMULATIONS AND RESULTS

Simulations required intensive use of the computer processor and about a time of 30 minutes requested for each. A model of each component has been recreated and then it was united in a single system. Specifically, it was analyzed how a stress repeated for 2000 times may influence the bone resorption in the peri-implant tissue. At the same time, the aim of the study was to analyze what is the weak point of the system and it has been recorded with the golden alloy black connection abutment screw. A force of 800 Nmm directed with 30° angulation for a cycle of 2000 times was applied. The Von Mises analysis was applied in the study in order to record the weak points of the system based on colors (red and yellow high stress) and around the bone tissue.

**Fig. (6):**
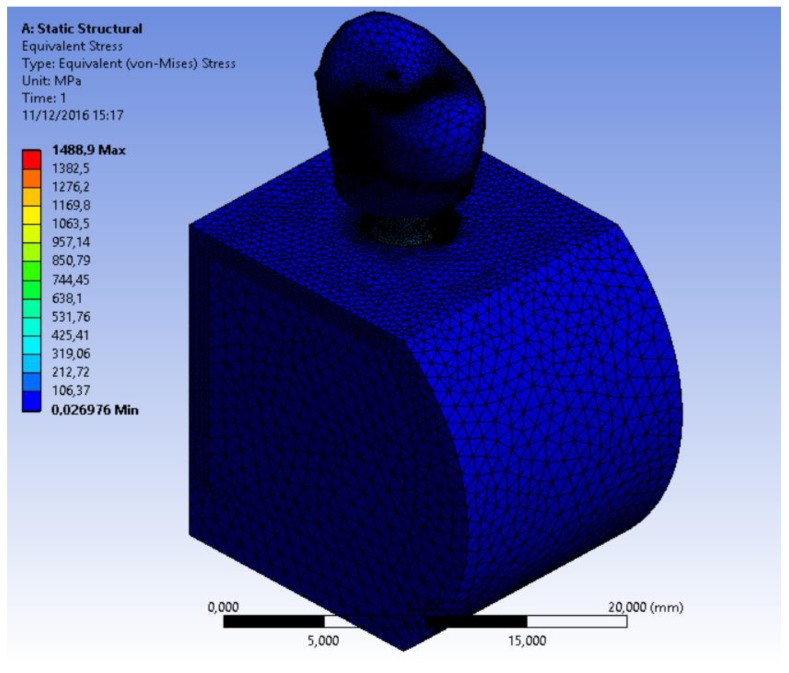
A stress equivalent was applied to the system and distributed to the model by the single point of the created mesh.

**Fig. (7):**
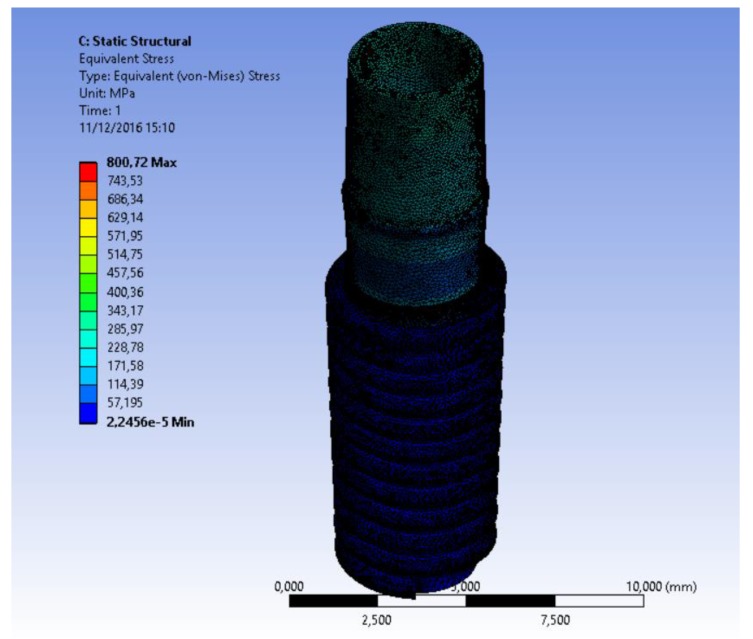
A stress equivalent was applied to the system and distributed to the fixture, abutment and abutment screw by the single point of the created mesh.

**Fig. (8):**
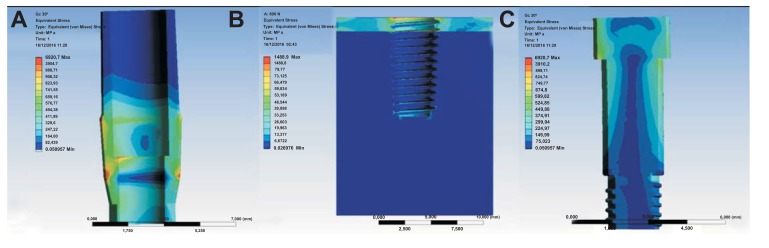
Different colors underline the 30° dynamic stress distribution area related to the used material. (A) The stress distributed to the abutment. Force is well tolerated; the weak point seems to be the area of the connection screw. (B) The fixture well tolerates the stress. Major stress is located at the neck of the implant. (C) The connection screw is the weak point of the system.

The results demonstrated the relationship between the loads applied to the system involving the geometrical characteristics of the materials, the constraints and deformations. The program, by using Von Mises analyses, expresses the results in the form of a chromatic scale of colors ranging from blue to red for the minimum values ​​to the maximum values. The values ​​represent those of the respective solution found. Firstly a static force of 800 Nmm was applied to the system and to each component of the fixture. Then a dynamic stress with 30° angulation was recreated by stimulating the components for a cyclic period of 2000 times (Figs. **6**-**8**).

## 4 DISCUSSION

The long-term clinical success of dental implant component loaded over bone tissue is a quite debated topic in the recent literature [1, 4, 7, 16-27]. The FEM and Von MISES methods of investigations have been recently applied for creating a virtual model of biomedical devices in order to analyse the strength distribution in the field of trauma, medicine and dentistry. The present research has been performed with a model created by using engineering software accordingly which has been recently published in the international literature [28-33].

Numerous papers investigated the application of a virtual three-dimensional model for evaluating stress distribution over orthodontic teeth movement. Hemanth et al. used an FEM model to evaluate the stress on periodontal ligament with intrusion and lingual root torque. Their analysis underlined that intrusive forces and lingual root torque produce stress at the root apex [34]. Similar results were found in the previous investigations for vertical tooth movement, where FEM was used. The rationale of the study was to highlight how various types of tooth movements like an intrusion and lingual root torque are associated with root resorption, especially with the incisors. However, the author concluded that FEM still remains an approximation study. The accuracy of the analysis is dependent on modeling the structure and material characterization as closely as possible to the actual. Therefore, according to the data of the present study, a virtual model is able to reflect all the strength and load related to complex forces like masticatory ones [4, 6, 8, 28, 31].

Others studies investigated how the biomechanics of complex systems, such as a tooth, needed the application of engineering research system and methods for evaluating stress movement and possible therapeutic options. Engineering machine and computers perform element analyses on such hard shape systems like dental devices with accuracy and preciseness. The FEM used in this research is an adequate method for the prediction of biomechanical behavior of the tooth under load [3, 9, 15, 25, 35].

The problem of the long time stability of the connection screw has been strongly investigated in the recent dental implants literature. Numerous brands of dental implants tried to create a new abutment connection in order to avoid the misfit and the possible loss of crown after repeated condition of the load. Conical, hexagonal, internal, and external or platform switching shape of dental implants is available for clinicians and practitioners. It is not possible to define the ideal one, however, all the connections should guarantee almost a long-term clinical success. Usually, once the abutment screw is tightened, a compression force is generated. Axial strengths located in the screw head are then transferred to the screw thread and implant contact surface. A clamping force able to fix the abutment to the fixture is obtained. The preload is equivalent to the clamping force in magnitude, and counteracts any force or load applied to the screw [32, 35]. The external force directed to the crown and then transferred to the dental components may result in the instability and micro-movement of the connection screw. Moreover, screw loosening may be an early warning of an inadequate biomechanical design and occlusal overloading. The model of the present study aimed to evaluate the stress related to connection screw with repeated stress with 30° angulation. The model offers a high guarantee for long-term stress distribution over dynamic force for several masticatory cycles. Von Mises analysed the result of good toleration about the stressed force to the entire investigated components.

## CONCLUSION

The main limitations of other previous published FEM studies are the impossibility of evaluating the dynamic force. In this investigation, the cycle of force repeated for several times (about 2000 times) in order to apply stress to all the system components, and not just applying force for once but reproducing a masticatory cycle. At least we can conclude that the fracture of the connection screws seems to be a protection for maintaining the fixture and crown, but at the same time detecting biomechanical problems.

## Figures and Tables

**Table 1:** Mechanical properties of the materials used in the study.

Mechanical Properties of the Materials						
–	Titanium Grade 4	Titanium Alloy Ti6Al4V	Cortical Bone	Cancellous Bone	Golden Alloy (Abutment Screw)	Zirconium Carbite
Density [kg/m^3^]	4510	4419	1800	1200	15000	6645
Young’s Modulus (E) [Mpa]	105000	108000	9600 (E_x_) 9600 (E_y_) 17800 (E_z_)*	144 (E_x_) 99 (E_y_) 344 (E_z_)*	95000	160670
Tangent Elastic Modulus (G) [Mpa]	-	-	3097 (G_xy_) 3510 (G_yz_) 3510 (G_xz_)	53 (G_xy_) 63 (G_yz_) 45 (G_xz_)	-	-
Poisson’s ratio (ν)	0,37	0,37	0,55 (ν_xy_) 0,3 (ν_yz_) 0,3(ν_xz_)	0,23 (ν_xy_) 0,11 (ν_yz_) 0,13 (ν_xz_)	0,35	0,35
Tensile Yield Strength (σ_y_) [Mpa]	485	830	115	32,4	800	939
Tensile Ultimate Strength (σ_u_)	550	900	133	37,5	855	430
Compressive Yield Strength (σ_y_) [Mpa]	485	830	182	51	700	758
Compressive Ultimate Strength(σ_u_) [Mpa]	-	-	195	55	-	0,195

## References

[1] Bramanti E., Cervino G., Lauritano F., Fiorillo L., D’Amico C., Sambataro S., Denaro D., Famà F., Ierardo G., Polimeni A., Cicciù M. (2017). FEM and von mises analysis on prosthetic crowns structural elements: Evaluation of different applied materials.. Sci. World J..

[2] van Staden R.C., Guan H., Johnson N.W., Loo Y.C., Meredith N. (2008). Step-wise analysis of the dental implant insertion process using the finite element technique.. Clin. Oral Implants Res..

[3] Kitagawa T., Tanimoto Y., Nemoto K., Aida M. (2005). Influence of cortical bone quality on stress distribution in bone around dental implant.. Dent. Mater. J..

[4] Cicciu M., Bramanti E., Matacena G., Guglielmino E., Risitano G. (2014). FEM evaluation of cemented-retained *versus* screw-retained dental implant single-tooth crown prosthesis.. Int. J. Clin. Exp. Med..

[5] Desai S.R., Karthikeyan I., Gaddale R. (2013). 3D finite element analysis of immediate loading of single wide *versus* double implants for replacing mandibular molar.. J. Indian Soc. Periodontol..

[6] Michailidis N., Karabinas G., Tsouknidas A., Maliaris G., Tsipas D., Koidis P. (2013). A FEM based endosteal implant simulation to determine the effect of peri-implant bone resorption on stress induced implant failure.. Biomed. Mater. Eng..

[7] Hoshaw S.J., Brunski J.B., Cochran G.V.B. (1994). Mechanical loading of Branemark implants affects interfacial bone modeling and remodeling.. Int. J. Oral Maxillofac. Implants.

[8] Lauritano F., Runci M., Cervino G. (2016). Three-dimensional evaluation of different prosthesis retention systems using finite element analysis and the Von Mises stress test.. Minerva Stomatol.

[9] Covani U., Ricci M., Tonelli P., Barone A. (2013). An evaluation of new designs in implant-abutment connections: A finite element method assessment.. Implant Dent..

[10] Roychowdhury A., Pal S. (2000). A 3-D FEM analysis of single and multiple screw-root dental implant fixed in a mandible.. Crit. Rev. Biomed. Eng..

[11] Moon H.J., Lee J.H., Choi K., Choi J.B., Koh C.S. (1997). Homogenized stress analysis in a dental implant system.. J. Med. Eng. Technol..

[12] Meriç G., Erkmen E., Kurt A., Eser A., Ozden A.U. (2012). Biomechanical comparison of two different collar structured implants supporting 3-unit fixed partial denture: A 3-D FEM study.. Acta Odontol. Scand..

[13] Cicciù M., Beretta M., Risitano G., Maiorana C. (2008). Cemented-retained *vs* screw-retained implant restorations: An investigation on 1939 dental implants.. Minerva Stomatol..

[14] Michailidis N., Karabinas G., Tsouknidas A., Maliaris G., Tsipas D., Koidis P. (2013). A FEM based endosteal implant simulation to determine the effect of peri-implant bone resorption on stress induced implant failure.. Biomed. Mater. Eng..

[15] Hoshaw S.J., Brunski J.B., Cochran G.V.B. (1994). Mechanical loading of Branemark implants affects interfacial bone modeling and remodeling.. Int. J. Oral Maxillofac. Implants.

[16] Holmgren E.P., Seckinger R.J., Kilgren L.M., Mante F. (1998). Evaluating parameters of osseointegrated dental implants using finite element analysis: A two-dimensional comparative study examining the effects of implant diameter, implant shape, and load direction.. J. Oral Implantol..

[17] Cicciù M., Risitano G., Maiorana C., Franceschini G. (2009). Parametric analysis of the strength in the “Toronto” osseous-prosthesis system.. Minerva Stomatol..

[18] Kitagawa T., Tanimoto Y., Odaki M., Nemoto K., Aida M. (2005). Influence of implant/abutment joint designs on abutment screw loosening in a dental implant system.. J. Biomed. Mater. Res. B Appl. Biomater..

[19] Boschian Pest L., Guidotti S., Pietrabissa R., Gagliani M. (2006). Stress distribution in a post-restored tooth using the three-dimensional finite element method.. J. Oral Rehabil..

[20] Roychowdhury A., Pal S., Saha S. (2000). Stress analysis of an artificial temporal mandibular joint.. Crit. Rev. Biomed. Eng..

[21] Van Staden R.C., Guan H., Loo Y.C. (2006). Application of the finite element method in dental implant research.. Comput. Methods Biomech. Biomed. Engin..

[22] Al-Sukhun J., Lindqvist C., Helenius M. (2007). Development of a three-dimensional finite element model of a human mandible containing endosseous dental implants. II. Variables affecting the predictive behavior of a finite element model of a human mandible.. J. Biomed. Mater. Res. A.

[23] Ernst C.P., Cohnen U., Stender E., Willershausen B. (2005). *In vitro* retentive strength of zirconium oxide ceramic crowns using different luting agents.. J. Prosthet. Dent..

[24] Proos K.A., Swain M.V., Ironside J., Steven G.P. (2003). Influence of core thickness on a restored crown of a first premolar using finite element analysis.. Int. J. Prosthodont..

[25] Shillingburg H.T., Hobo S., Whitsett L.D., Jacobi R., Brackett S.E. (1997). Fundamentals of fixed prosthodontics..

[26] Vult von Steyern P., Carlson P., Nilner K. (2005). All-ceramic fixed partial dentures designed according to the DC-Zirkon technique. A 2-year clinical study.. J. Oral Rehabil..

[27] Kelly J.R. (1999). Clinically relevant approach to failure testing of all-ceramic restorations.. J. Prosthet. Dent..

[28] Lawn B.R., Pajares A., Zhang Y., Deng Y., Polack M.A., Lloyd I.K., Rekow E.D., Thompson V.P. (2004). Materials design in the performance of all-ceramic crowns.. Biomaterials.

[29] Bergkvist G., Simonsson K., Rydberg K., Johansson F., Dérand T. (2008). A finite element analysis of stress distribution in bone tissue surrounding uncoupled or splinted dental implants.. Clin. Implant Dent. Relat. Res..

[30] Sannino G., Barlattani A. (2013). Mechanical evaluation of an implant-abutment self-locking taper connection: Finite element analysis and experimental tests.. Int. J. Oral Maxillofac. Implants.

[31] Bruna-Rosso C., Arnoux P-J., Bianco R-J., Godio-Raboutet Y., Fradet L., Aubin C-É. (2016). Finite element analysis of sacroiliac joint fixation under compression loads.. Int. J. Spine Surg..

[32] Cicciù M., Cervino G., Bramanti E., Lauritano F., Lo Gudice G., Scappaticci L., Rapparini A., Guglielmino E., Risitano G. (2015). FEM analysis of mandibular prosthetic overdenture supported by dental implants: Evaluation of different retention methods.. Comput. Math. Methods Med..

[33] Lauritano F., Runci M., Cervino G., Fiorillo L., Bramanti E., Cicciù M. (2016). Three-dimensional evaluation of different prosthesis retention systems using finite element analysis and the Von Mises stress test.. Minerva Stomatol..

[34] Hemanth M., Deoli S., Raghuveer H.P. (2015). deoli S, Raghuveer HP, Rani MS, Hegde C, Vedavathi B. Stress induced in the periodontal ligament under orthodontic loading (Part I): A finite element method study using linear analysis.. J. Int. Oral Health.

[35] Paepoemsin T., Reichart P.A., Chaijareenont P., Strietzel F.P., Khongkhunthian P. (2016). Removal torque evaluation of three different abutment screws for single implant restorations after mechanical cyclic loading.. Oral Implantol. (Rome).

